# Combined effects of sodium-glucose cotransporter 2 inhibitor and angiotensin receptor-neprilysin inhibitor on renal function in cardiovascular disease patients with type 2 diabetes mellitus: a retrospective cohort study

**DOI:** 10.3389/fendo.2023.1326611

**Published:** 2024-01-11

**Authors:** Ling Xu, Bo Chen, Hua Zhang, Dan Zhu

**Affiliations:** ^1^ Department of Cardiology, Peking University Third Hospital, NHC Key, Laboratory of Cardiovascular Molecular Biology and Regulatory Peptides, Peking University, Beijing, China; ^2^ Research Center of Clinical Epidemiology, Peking University Third Hospital, Peking University, Beijing, China

**Keywords:** ARNI, SGLT-2 inhibitor, combination treatment, renal function, cardiovascular disease, diabetes

## Abstract

**Background:**

Angiotensin receptor/neprilysin inhibitor (ARNI) and sodium-glucose cotransporter 2 inhibitor (SGLT2i) have shown a significant protective role against cardiovascular diseases and type 2 diabetes mellitus (T2DM), and there is a growing proportion of patients who are undergoing combined therapy with the two drugs. However, the effect of this combination treatment on renal function has not yet been determined.

**Methods:**

This study included 539 patients who were diagnosed with cardiovascular disease combined with T2DM. According to the use of SGLT2i and ARNI, patients were divided into the combination treatment group, SGLT2i group, ARNI group and control group. Primary outcomes were serum creatinine (Scr) and estimated glomerular filtration rate (eGFR) changes in the 6th month and 12th month.

**Results:**

In the ARNI group, no significant changes in Scr or eGFR were observed during the follow-up period, while the above indicators showed a trend of deterioration in the other three groups. The univariate analysis results showed that at 6 months of follow-up, the renal function indicators of patients treated with ARNI (either alone or in combination) were better than those treated with SGLT2i alone. After 12 months of follow-up, the Scr results were the same as before, while the difference in eGFR between groups disappeared. After multivariate analysis, in terms of delaying the progression of Scr, the ARNI group was superior to the other groups at the end of follow-up. No significant difference in eGFR was observed between groups during follow-up.

**Conclusion:**

In patients with cardiovascular disease and T2DM, combination therapy with ARNI and SGLT2i did not show an advantage over monotherapy in delaying renal insufficiency progression, and renal function seems to be better preserved in patients treated with ARNI alone.

**Clinical trial registration:**

clinicaltrials.gov, identifier NCT05922852.

## 1 Introduction

Cardiovascular disease (CVD), due to its high risk of morbidity and mortality, remains a major public health issue in China and even worldwide. Type 2 diabetes mellitus (T2DM) is an established major risk factor for CVD (1). The China Kadoorie Biobank (CKB) followed up with 510,000 adults for a duration of 7 years. The findings revealed that diabetes is associated with a 1.5- to 2.5-fold increase in the risks of cardiovascular mortality, incident ischemic heart disease, and ischemic stroke. Additionally, diabetes accounted for approximately 500,000 cardiovascular deaths annually in China (2). Many drugs have been used to prevent and delay the progression of cardiovascular disease in T2DM patients, among which a new drug, sodium-glucose cotransporter 2 inhibitor (SGLT2i), has gradually gained attention due to its combined improvement of cardiovascular outcomes and long-term prognosis (3, 4). Therefore, in recent years, various international guidelines have recommended SGLT2i as a basic medication for patients with diabetes with CVD or at risk of CVD (5–8). Angiotensin receptor-neprilysin inhibitor (ARNI) is another commonly used drug for patients with CVD because of its significant improvement in long-term prognosis (8, 9). In view of its additional metabolic benefits, ARNI is particularly beneficial for patients complicated with diabetes (10, 11).

As diabetes is closely related to CVD and the above two drugs are increasingly used in clinical practice, the proportion of patients taking them in combination is gradually increasing. However, there is no clear conclusion on the impact of the combined treatment regimens on renal function in cardiovascular disease patients, especially those with T2DM. These patients usually have varying degrees of renal function impairment. And the interaction between CVD and renal dysfunction can exacerbate each other when T2DM is present, resulting in a higher morbidity and mortality (12). Previous studies have investigated the effects of the above two drugs on renal function, and found that both ARNI and SGLT2i could delay the deterioration of renal function to a certain extent in long-term follow-up, but some other studies have shown different results (13, 14). As renal function is one of the important prognostic factors, and currently few studies have evaluated the effect of combining the above two drugs on renal function compared with either agent alone. This study intends to collect data on patients with cardiovascular disease and T2DM and explore the safety and efficacy of the combined use of ARNI and SGLT2i on renal function in the real world to provide a basis for better guiding the future clinical application of these drugs.

## 2 Methods

### 2.1 Study design

This was a retrospective cohort study that included patients who were diagnosed with cardiovascular disease and combined with T2DM in the Cardiology Department, Peking University Third Hospital from January 2018 to November 2022. According to the medication used after discharge, patients were divided into the SGLT2i and ARNI combination treatment group (CT group), SGLT2i group, and ARNI group, and patients who did not use the above drugs were included in the control group. We followed up the included patients for 1 year and collected data on renal function-related indicators for subsequent analysis.

### 2.2 Participants

Our study included patients aged 18 years or older who were diagnosed with cardiovascular disease (including hypertension, coronary heart disease, heart failure, etc., see **Table 1** for details) combined with an established diagnosis of T2DM (9) in the Cardiology Department, Peking University Third Hospital from January 2018 to November 2022.

**Table 1:** Baseline information.

	ARNI group (N=64)	SGLT2i group(N=111)	CT group (N=71)	Control group (N=293)	P value
General Condition					
**Gender[Male, N(%)]**	42(65.6)	73(65.8)	54(76.1)	176(60.1)	0.083
**Age(y)**	67.5 ± 5.4	65.3 ± 6.2	65.8 ± 6.0	67.3 ± 6.7	0.085
**Height(cm)**	166.8 ± 7.2	167.0 ± 7.9	168.9 ± 8.9	166.0 ± 8.8	0.082
**Weight(kg)**	69.7 ± 11.4a	73.9 ± 16.8b	78.9 ± 16.4b	70.1 ± 13.1a	0.020
Past Medical History					
**Hypertension, N(%)**	55(85.9)a,b	82(73.9)b	52(73.2)b	273(92.3)a	<0.001
**Hyperlipidemia, N(%)**	35(54.7)	67(60.4)	36(50.7)	160(54.6)	0.610
**CHD,N(%)**	49(76.6)	106(95.5)	64(90.1)	255(87.0)	0.002
**Stroke,N(%)**	16(25.0)	19(17.1)	21(29.6)	55(18.8)	0.128
**Heart Failure,N(%)**	39(60.9)a	3(2.7)b	25(35.2)c	39(13.3)d	<0.001
**CKD,N(%)**	10(15.6)a	4(3.6)b	8(11.3)a,b	32(10.9)a,b	0.053
Medications					
**AECI/ARB,N(%)**	0(0)a	55(49.5)b	0(0)a	290(99.0)c	<0.001
**GLP-1RA,N(%)**	1(1.6)a	15(13.5)b	10(14.1)b	23(7.8)a,b	0.021
**Statins,N(%)**	58(90.6)	108(97.3)	68(95.8)	271(92.5)	0.196
**β-blocker,N(%)**	42(65.6)	75(67.6)	58(81.7)	211(72.0)	0.133
**CCBs,N(%)**	22(34.4)a,b	34(30.6)b	25(35.2)a,b	154(52.6)a	<0.001
**Spirolactone,N(%)**	26(40.6)a	6(5.4)b	26(36.6)a	15(5.1)b	<0.001
Baseline Biomakers(Serum sample)					
**Uric acid(μmol/l)**	428.2 ± 152.1a	365.8 ± 117.0c	382.6 ± 126.6c	401.1 ± 114.2b	0.006
**Urea Nitrogen(mmol/l)**	9.0 ± 4.1a	6.8 ± 2.8c	8.6 ± 3.6b	7.2 ± 2.9c	<0.001
**Creatinine(μmol/l)**	104.7 ± 52.5a	84.6 ± 30.5c	105.1 ± 41.5a	98.9 ± 42.7b	<0.001
**eGFR(ml/min/1.73m^2^)**	64.2 ± 20.9a	81.5 ± 20.0c	69.8 ± 23.9b	69.5 ± 21.3b	<0.001
**HbA1C(%)**	6.8(6.4, 7.9)a	8.0(7.1, 9.0)c	7.9(6.7, 8.9)c	7.3(6.7, 8.3)b	0.001
**LDL-c(mmol/l)**	2.5 ± 0.9	2.4 ± 1.1	2.4 ± 1.1	2.3 ± 0.9	0.620
**NT-proBNP(pg/ml)**	1447.0(435.3, 4105.8)a	172.0(48.0, 694.5)c	1556.5(335.0, 3811.8)a	281.0(93.0, 1141.5)b	<0.001

Normally distributed data are expressed as the mean ± standard deviation, and nonnormally distributed data are expressed as the median (interquartile range).

Each subscript letter indicates a subset of the group category whose column proportions are not significantly different from each other at the 0.05 level.

ARNI, angiotensin receptor-neprilysin inhibitor; SGLT2i, sodium-glucose cotransporter 2 inhibitors; CT group, combination treatment group; CHD, coronary heart disease; CKD, chronic kidney disease; ACEI, angiotensin converting enzyme inhibitor; ARB, angiotensin receptor blocker; CCB, calcium channel blocker; GLP-1RA, glucagon-like peptide-1 receptor agonist; eGFR, estimated glomerular filtration rate; LDL-c, low-density lipoprotein cholesterol.

**Figure 1:**
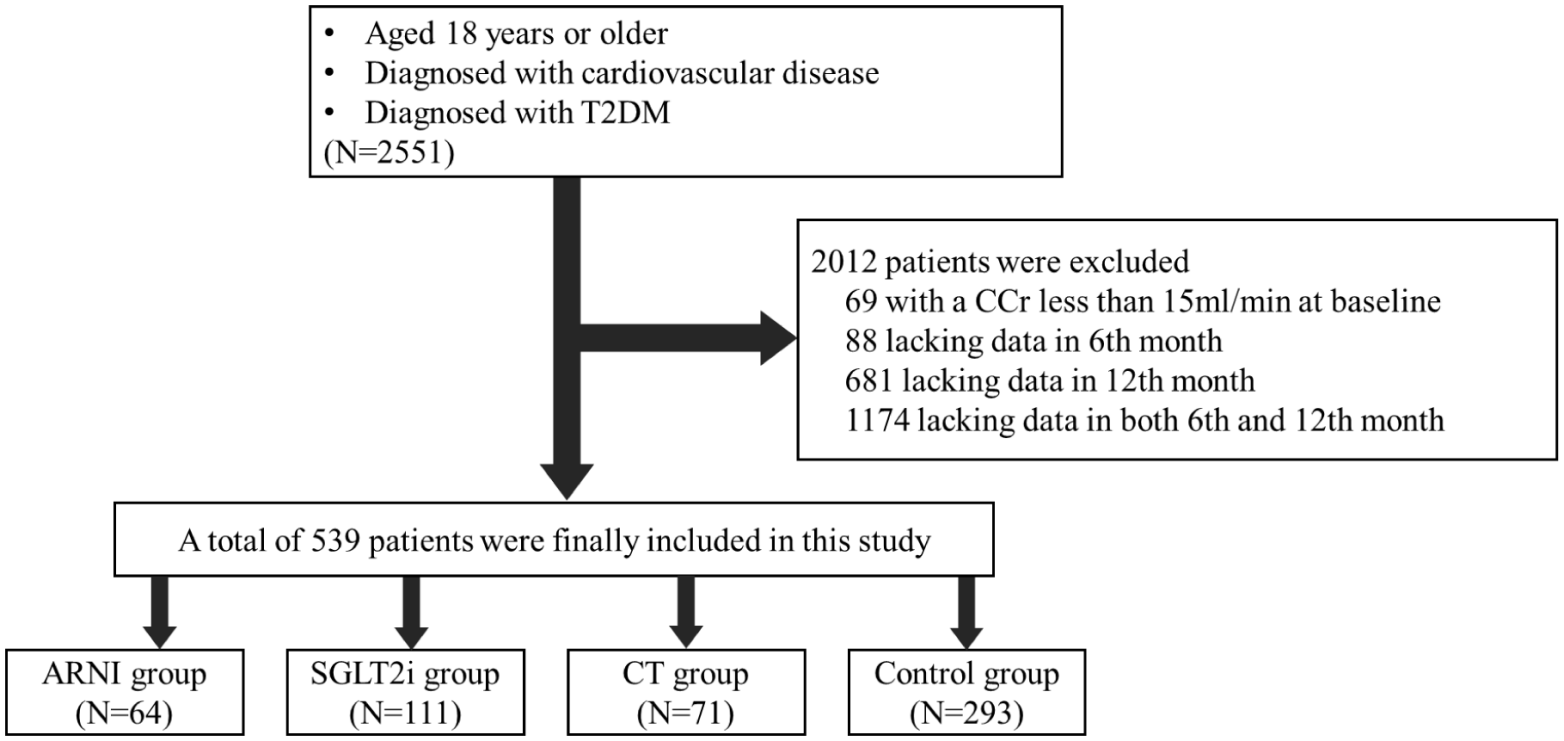
Enrollment and Exclusion. This study included patients who were diagnosed with cardiovascular disease and combined with T2DM in the Cardiology Department, Peking University Third Hospital from January 2018 to November 2022. After screening by inclusion and exclusion criteria, 539 patients were finally included in the follow-up cohort.

The exclusion criteria were as follows: 1) estimated glomerular filtration rate (eGFR) less than 15 ml/min/1.73m^2^) at discharge, as these patients were not recommended for any medication above; 2) clinical diagnosis of various acute and chronic urinary tract infections; 3) various serious infectious diseases; 4) acute coronary syndrome and unstable heart failure; 5) acute complications of diabetes mellitus, such as diabetic ketoacidosis, hyperosmolar coma and hypoglycemia; 6) end-stage malignancy; 7) pregnancy and lactation; and 8) lack of data during follow-up (see **Figure 1** for details).

### 2.3 Data and clinical outcomes

Data on demographic and laboratory characteristics at baseline, as well as data on renal outcomes during follow-up, were retrospectively abstracted from computerized medical records by clinical staff. The outcomes included serum creatinine (Scr) and eGFR changes at the 6th month and 12th month of follow-up in each group and differences in Scr and eGFR between combined therapy and monotherapy with ARNI/SGLT2i at each time point of follow-up.

### 2.4 Statistical analysis

Categorical data were summarized as numbers and percentages, which were then compared using the chi-square or Fisher’s exact test. P-P plots were used to evaluate the distribution of all continuous variables. Nonnormally distributed continuous variables were described as the median and interquartile range and were tested using the Mann−Whitney U test. Normally distributed continuous variables that did not differ between groups at baseline were described as the mean ± SD and were tested using independent t tests. For those with significant differences in distribution between groups at baseline, we used analysis of covariance (ANCOVA) to improve data comparability. The results calculated using ANCOVA were expressed as the mean and 95% confidence interval (CI).

All analyses were conducted using SPSS Statistics for Windows, Version 26.0 (Armonk, NY: IBM Corp). Statistical tests were two-sided, and significance was set at a p value < 0.05.

## 3 Results

### 3.1 Baseline information

A total of 539 subjects were included in the follow-up cohort, including 64 patients receiving ARNI alone, 111 patients receiving SGLT2i alone, 71 patients receiving combination treatment and the rest were included in the control group. The baseline information of the patients between the groups was not consistent. Patients who chose ARNI had a higher rate of heart failure and worse basic renal function, while patients who chose SGLT2i had worse basic glycemic control but better basic renal function. In view of the differences in underlying diseases, there are corresponding differences in the drug application of each group of patients. Besides, it’s should be noted that almost 99% of control patients were treated with angiotensin-converting enzyme inhibitors (ACEI) or angiotensin receptor blockers (ARB) and approximately 50% of SGLT2i monotherapy patients received ACEI/ARB treatment (see **Table 1** for details).

### 3.2 Changes in renal function

#### 3.2.1 Changes in renal function in each group during follow-up

Overall, the renal function of the subjects showed a trend of deterioration during the follow-up period. However, there were no significant changes in either Scr (12th month vs. Baseline: 112.8 ± 52.9 vs. 104.7 ± 52.5, P=0.210) or eGFR (12th month vs. Baseline: 61.7 ± 22.5 vs. 64.2 ± 20.9, P=0.176) during follow-up in patients taking ARNI alone compared with baseline, while the other three groups showed significant deterioration of renal function during the follow-up period, but the difference mainly appeared in the first 6 months of follow-up in SGLT2i group and CT group (SGLT2i group-Scr: 6th month vs. Baseline: 93.1 ± 53.4 vs. 84.6 ± 30.5, P=0.006; CT group-Scr: 6th month vs. Baseline: 112.9 ± 37.4 vs. 105.1 ± 41.5, P=0.004; SGLT2i group-eGFR: 6th month vs. Baseline:76.6 ± 19.2 vs. 81.5 ± 20.0, P<0.001; CT group-eGFR: 6th month vs. Baseline: 61.3 ± 20.5 vs. 69.8 ± 23.9, P<0.001), and there was no further deterioration of Scr or eGFR during the last 6 months of follow-up (SGLT2i group-Scr: 12th month vs. 6th month: 94.4 ± 55.1 vs. 93.1 ± 53.4, P=0.401; CT group-Scr: 12th month vs. 6th month:114.7 ± 48.2 vs. 112.9 ± 37.4, P=0.563; SGLT2i group-eGFR: 12th month vs. 6th month: 76.4 ± 20.3 vs. 76.6 ± 19.2, P=0.538; CT group-eGFR: 12th month vs. 6th month: 61.7 ± 21.0 vs. 61.3 ± 20.5, P=0.637). However, in Control group, renal function indicators declined significantly in both the first and the later 6 months (Scr: 6th month vs. Baseline: 109.0 ± 43.8 vs. 98.9 ± 42.7, P<0.001; 12th month vs. 6th month: 115.0 ± 64.9 vs. 109.0 ± 43.8, P=0.026; eGFR: 6th month vs. Baseline: 62.7 ± 21.2 vs. 67.9 ± 23.1, P<0.001; 12th month vs. 6th month: 61.1 ± 21.8 vs. 62.7 ± 21.2, P=0.018) (see **Table 2** for details).

**Table 2:** Changes in renal function indicators during the follow-up.

	Serum Creatinine (μmol/l)			Estimated Glomerular Filtration Rate (ml/min/1.73m^2^)		
	Baseline	Followup-6M	Followup-12M	Baseline	Followup-6M	Followup-12M
**ARNI group**	104.7 ± 52.5	110.9 ± 36.4	112.8 ± 52.9	64.2 ± 20.9	59.7 ± 22.1	61.7 ± 22.5
**SGLT2i group**	84.6 ± 30.5a	93.1 ± 53.4b	94.4 ± 55.1b	81.5 ± 20.0a	76.6 ± 19.2b	76.4 ± 20.3b
**CT group**	105.1 ± 41.5a	112.9 ± 37.4b	114.7 ± 48.2b	69.8 ± 23.9a	61.3 ± 20.5b	61.7 ± 21.0b
**Control group**	98.9 ± 42.7a	109.0 ± 43.8b	115.0 ± 64.9b	67.9 ± 23.1a	62.7 ± 21.2b	61.1 ± 21.8b

Data are expressed as the mean ± standard deviation. Each subscript letter indicates a subset of the group category whose column proportions are not significantly different from each other at the 0.05 level.

ARNI, angiotensin receptor-neprilysin inhibitor; SGLT2i, sodium-glucose cotransporter 2 inhibitors; CT group, combination treatment group.

#### 3.2.2 Differences in renal function between groups at each follow-up point

In this part, we mainly compared the three groups receiving the target drugs. As mentioned above, there were differences in renal function among the groups at baseline, and the basic renal function of the patients receiving SGLT2i alone was better than that of the other two groups. In order to balance the baseline differences in renal function indicators among the groups, we applied covariance analysis and included baseline Scr or eGFR as covariates for univariate analysis. In terms of the comparison of Scr levels, patients who used ARNI (either alone or in combination treatment group) were better than patients treated with SGLT2i alone during follow-up (ARNI group vs. SGLT2i group vs. CT group: 6th Month: 106.8(100.3,113.2) vs. 109.7(104.5,114.9) vs. 105.8(99.6, 112.0), P=0.001; 12th Month:108.1(99.2,116.9) vs. 111.4(104.3,118.5) vs.106.9(98.4, 115.4), P=0.001). As for the comparison of eGFR, the combination treatment group was lower than the SGLT2i group at 6 months of follow-up (ARNI group vs. SGLT2i group vs. CT group: 66.9(63.5,70.2) vs. 70.6(68.1, 73.1) vs. 65.8(62.8, 68.9), P=0.049), but this difference disappeared at 12 months of follow-up (P=0.225) (see **Table 3** for details).

**Table 3:** Intergroup differences in renal function indicators at each follow-up point.

	ARNI group (N=64)	SGLT2i group(N=111)	CT group (N=71)	P value
Serum Creatinine (μmol/l)				
**Baseline**	104.7(91.6, 117.8)	84.6(78.8, 90.3)	105.1(95.2, 114.9)	0.001
**Follow-up 6M**	106.8(100.3, 113.2)b	109.7(104.5, 114.9)a	105.8(99.6, 112.0)b	0.001
**Follow-up 12M**	108.1(99.2, 116.9)a,b	111.4(104.3, 118.5)a	106.9(98.4, 115.4)b	0.001
Estimated Glomerular Filtration Rate (ml/min/1.73m^2^)				
**Baseline**	64.2(58.9, 69.5)	81.5(77.7,85.2)	69.8(64.1, 75.5)	0.001
**Follow-up 6M**	66.9(63.5, 70.2)a,b	70.6(68.1, 73.1)a	65.8(62.8, 68.9)b	0.049
**Follow-up 12M**	68.5(64.9, 72.0)	69.7(66.9, 72.5)	66.0(62.6, 69.4)	0.255

Univariate analysis was performed with baseline Scr or eGFR included as covariates by covariance analysis. Data were expressed as the mean (95% CI).

Each subscript letter indicates a subset of the group category whose column proportions are not significantly different from each other at the 0.05 level.

ARNI, angiotensin receptor-neprilysin inhibitor; SGLT2i, sodium-glucose cotransporter 2 inhibitors; CT group, combination treatment group.

**Figure 2:**
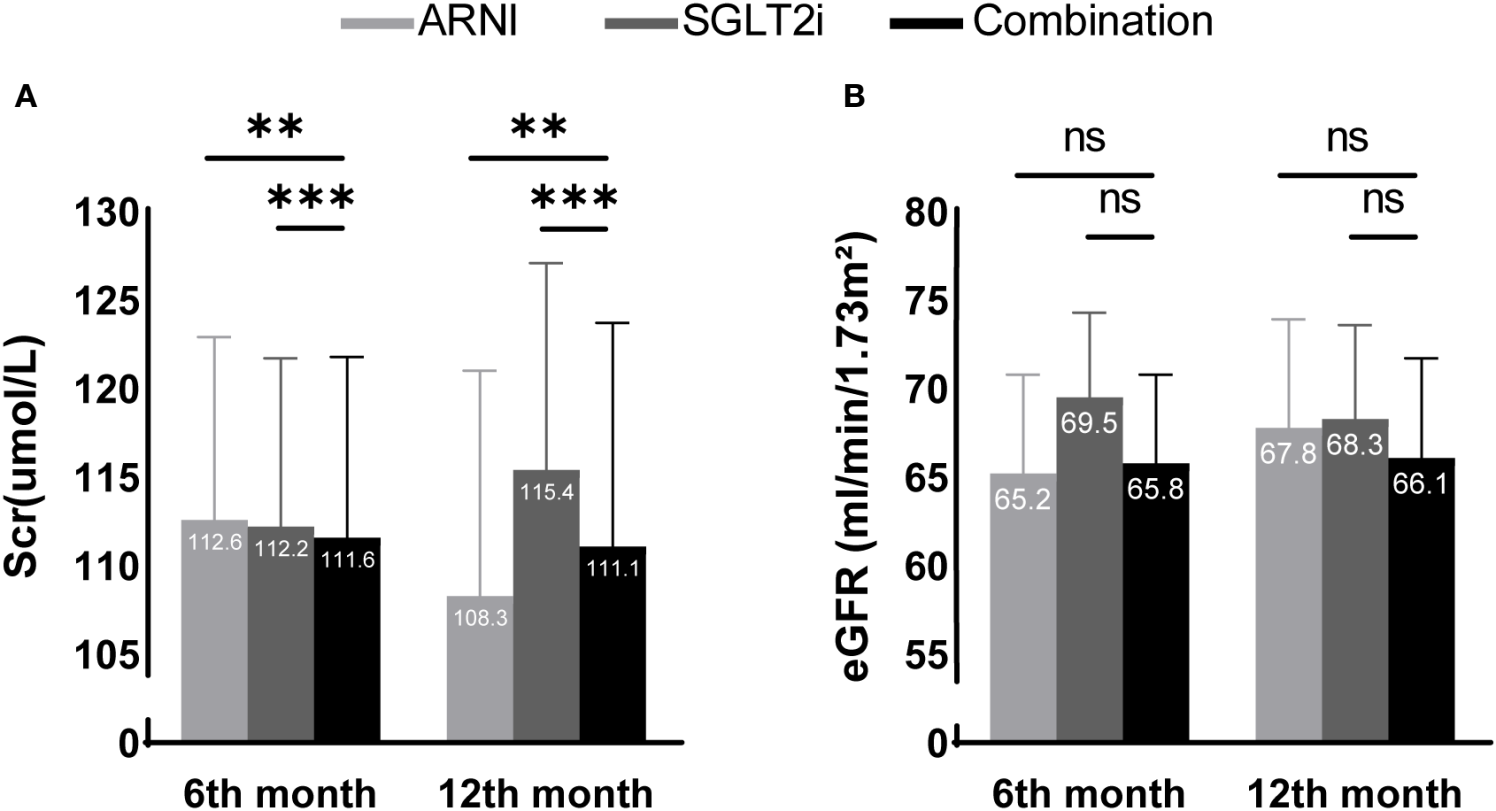
Scr **(A)** and eGFR **(B)** differences between groups adjusted after multivariate analysis. ** and *** present a significance of <0.01 and <0.001 compared with the combination treatment group, respectively. ns, no significance. In multivariate analysis, the combination treatment group was significantly better than the other two groups in Scr after 6 months of follow-up, but the advantage of the ARNI group was more significant at 12 months of follow-up. Using eGFR as the evaluation index, no differences were shown among the three groups during the follow-up period.

We then further conducted multivariate analysis. Combined with the results of baseline information analysis, we included baseline Scr or eGFR, body weight, baseline HbA1c level and NT-proBNP level, history of hypertension, heart failure, coronary heart disease, use of ACEI/ARB, and chronic kidney disease into the analysis as covariates. In terms of Scr, although the combination treatment group seemed to show an advantage at 6 months of follow-up (ARNI group vs. CT group: 112.6 (102.4,122.9) vs. 111.6 (101.5,121.8), P=0.003; SGLT2i group vs. CT group: 112.2 (102.8,121.7) vs. 111.6 (101.5,121.8), P<0.001), the ARNI group was superior to the other two groups at 12 months of follow-up(ARNI group vs. CT group: 108.3 (95.6, 121.0) vs. 111.1 (98.6, 123.7), P=0.003; SGLT2i group vs. CT group: 115.4 (103.6, 127.1) vs. 111.1 (98.6, 123.7), P<0.001). No significant difference in eGFR was observed between groups during follow-up (see **Table 4** and **Figure 2** for details).

**Table 4:** Multivariate analysis of the distribution of renal function indexes among the groups.

	Followup-6M		Followup-12M	
	Mean(95%CI)	P value(Compared to CT group)	Mean(95%CI)	P value(Compared to CT group)
Serum Creatinine (μmol/l)				
**ARNI group**	112.6(102.4,122.9)	0.003	108.3(95.6, 121.0)	0.003
**SGLT2i group**	112.2(102.8,121.7)	<0.001	115.4(103.6, 127.1)	<0.001
**CT group**	111.6(101.5,121.8)	—	111.1(98.6, 123.7)	—
Estimated Glomerular Filtration Rate (ml/min/1.73m^2^)				
**ARNI group**	65.2(59.6, 70.8)	0.294	67.8(61.6, 73.9)	0.547
**SGLT2i group**	69.5(64.8, 74.3)	0.617	68.3(63.0, 73.6)	0.412
**CT group**	65.8(60.8, 70.8)	—	66.1(60.5, 71.7)	—

Multivariate analysis was performed with baseline Scr or eGFR, body weight, baseline HbA1c level and NT-proBNP level, history of hypertension, heart failure, coronary heart disease, use of ACEI/ARB, and chronic kidney disease included as covariates by covariance analysis. Data were expressed as the mean (95% CI).

ARNI, angiotensin receptor-neprilysin inhibitor; SGLT2i, sodium-glucose cotransporter 2 inhibitors; CT group, combination treatment group.

## 4 Discussion

Through this retrospective cohort study, we found that on the basis of existing drug therapy, renal function in cardiovascular disease patients with T2DM still showed an overall downward trend during follow-up. Therefore, the optimal drug therapy for such patients needs to be further clarified. In terms of delaying the progression of renal function, the results of the group comparison did not find an advantage of the combination of ARNI and SGLT2i over monotherapy, and the treatment regimen of ARNI alone seemed to be better.

A large number of studies have confirmed the excellent role of ARNI and SGLT2i in improving cardiovascular outcomes (10, 11) and recently found that the above drugs also have a certain improvement effect on long-term kidney outcomes (13–17). Therefore, ARNI and SGLT2i are now widely used in patients with cardiovascular diseases, and there are not a few people who use them in combination. Renal function is an important factor affecting the long-term prognosis of patients with cardiovascular disease. From the mechanism of drug action, the advantages of combined drug use seem to be based on evidence: 1) The mechanisms by which the two drugs reduce intraglomerular pressure are different and may exert complementary effects; 2) The natriuretic and diuretic effect of SGLT2i may activate the renin-angiotensin-aldosterone (RAAS) system when used alone, but combined use with ARNI may offset this effect; 3) This combination may synergistically block sodium-hydrogen exchanger activity, and the latter activity is stimulated by hyperglycemia, hyperinsulinemia and adipokines, which may promote the occurrence and development of glomerular hyperfiltration and diabetic nephropathy, and at the same time play an important role in the pathophysiology of heart failure (18). Therefore, it is the focus of this study to explore whether the combination of two drugs can delay the deterioration of renal function more significantly than a single drug in the real world.

According to the results of this study, renal function seems to be better preserved in patients treated with ARNI alone. Previous studies have actually explored the many benefits of SGLT2i on renal function. The main mechanism of SGLT2i is to block SGLT2 in the proximal tubule of the kidney to increase the excretion of glucose and sodium in the urine, which can activate renal tubule-glomerular feedback (TGF), thus alleviating the glomerular hyperfiltration state. Meanwhile, the excretion of glucose reduces glucose overload in proximal tubule cells. Thus, the apoptosis and atrophy of renal tubules are reduced. Other studies have found that SGLT2i can significantly inhibit inflammation and fibrosis (19, 20). Therefore, the SGLT2i improves the long-term renal prognosis through multiple mechanisms.

However, in this study, no positive effect of SGLT2i on renal function was observed during the 1-year follow-up period using Scr and eGFR as evaluation indicators, either alone or in combination with ARNI. In patients with diabetic nephropathy, the glomerular filtration function of pathological glomeruli decreases; therefore, in order to maintain overall renal filtration function, the remaining functioning glomeruli require compensatory hyperfiltration (21). It has been found that SGLT2i can not only shrink the afferent arteriole but also dilate the efferent arterioles, thereby alleviating the hyperfiltration state, but at the same time weakening the overall filtration function of the kidney, especially in patients who have been treated with ARNI (efferent arterioles have been dilated by ARNI). As mentioned above, for patients treated with SGLT2i alone, their natriuretic and blood volume-reducing effects can activate the RAAS, and this effect is more prominent in patients with hemodynamic instability or hypovolemia. In addition, the inhibition of SGLT2 leads to an increase in solute flow into the distal tubules, which increases the transport load of the distal tubules, while the renal medulla itself has limited oxygen uptake capacity. The above process may induce hypoxia injury of the renal medulla. Meanwhile, the glucose gathered in the distal tubules can further aggravate renal tubule injury by generating fructose and uric acid, inducing oxidative stress and inflammation (21, 22). Therefore, the effect of SGLT2i in improving renal function may be limited due to the above mentioned mechanism.

The limitations of this study are as follows. First, Perkovic et al. published a study that combined renin–angiotensin system blockade and canagliflozin in patients with diabetic nephropathy. During nearly 3 years of follow-up, it was found that the combination treatment could slow down the deterioration of eGFR compared with renin–angiotensin system blockade alone. However, in the first year of treatment, eGFR decreased more significantly in the combination treatment group. Meanwhile, in the subgroup analysis, it was found that patients with better basic renal function and less urinary protein had the least improvement in renal function (23), while the mean eGFR of patients included in this study was 64.2-81.5 ml/min/1.73m^2^), and the renal function in the SGLT2i group at baseline was better. As ARNI partially share similar mechanisms to ACEI/ARB, our research results might be affected by the fact that the subjects included were not extensive enough and the follow-up time was relatively short. Second, previous studies suggest that for patients with ARNI combined with SGLT2i, the sequence of use of the two drugs may affect the change trend of renal function. For those who add ARNI on the basis of SGLT2i, renal function is more inclined to improve, while the reverse may worsen (24). The combination treatment group in this study was not further subdivided according to the order of medication, which may affect the results. Finally, combining the information from the FDA and the results of a recent meta-analysis, different types of SGLT2i may have different effects on renal function (25, 26). The patients in this study were not further analyzed based on SGLT2i types, which may also affect the results.

## 5 Conclusion

This retrospective cohort study included cardiovascular disease patients with T2DM, grouped patients according to the use of SGLT2i and ARNI, and followed up for 1 year. The results did not show that combination therapy could delay renal function progression better than monotherapy. In the future, it may be necessary to expand the scope of subjects, extend the follow-up time, and further demonstrate the problem through RCTs or meta-analyses.

## Data availability statement

The raw data supporting the conclusions of this article will be made available by the authors, without undue reservation.

## Ethics statement

The studies involving humans were approved by The Medical Science Research Ethics Committee Peking University Third Hospital. The studies were conducted in accordance with the local legislation and institutional requirements. The human samples used in this study were acquired from a by- product of routine care or industry. Written informed consent for participation was not required from the participants or the participants’ legal guardians/next of kin in accordance with the national legislation and institutional requirements.

## Author contributions

LX: Data curation, Formal Analysis, Investigation, Project administration, Visualization, Writing – original draft, Writing – review & editing. BC: Data curation, Formal Analysis, Investigation, Project administration, Visualization, Writing – original draft, Writing – review & editing. HZ: Methodology, Writing – review & editing. DZ: Conceptualization, Funding acquisition, Resources, Supervision, Writing – review & editing.
